# Chronic Disease Among the Indigenous Population of Central Australia

**Published:** 2006-03-15

**Authors:** Gill O'Connor

**Affiliations:** Workforce Strategy & Clinical Learning Team, Nursing Clinical Team, Alice Springs Hospital, Alice Springs, Australia

## To the Editor:

I educate and consult with health care providers in chronic disease prevention, self-management, and screening. I also work with the Aboriginal Cultural Awareness Program team teaching new staff about Australian Aboriginal culture from a "white-fellas" perspective. The indigenous population of central Australia is very traditional and maintains cultural practices. There are 10 dominant indigenous languages spoken in central Australia. Chronic diseases — including renal disease, insulin-resistant diabetes, respiratory disease (especially bronchiectasis), heart disease and hypertension, and mental health — are in plague proportions. High alcohol intake has lead to increased liver disease, and substance abuse such as petrol sniffing has increased the need for respite care among young Aboriginal people. The population of central Australia (area of 1.5 million sq km) is 45,000 people, and approximately 14,000 are of Aboriginal descent. Aboriginal people of central Australia are very traditional and maintain cultural practices. There are 25 central Australian indigenous languages spoken, and the five dominant languages spoken are Arrernte, Anmatjyerre, Alwyare, Pitjantjatarra, Luritja, and Walpiri. Alice Springs (population 26,000) is in the Arrernte region, and there are five dialects of Arrernte spoken among 1800 people.

I started nursing in Alice Springs in 1980, and many of the babies with gastroenteritis and malnutrition I nursed then I saw in later years with end-stage chronic disease. I have attended the funerals of some of these children who only lived to their late teens or early 20s. Over the years, many programs have been set up, committees and working parties have set agendas, and projects have been developed to tackle the chronic disease issues we face, but unless these programs involve the Aboriginal people (so that the correct language is used, concepts are ensured, appropriate health literacy is used, and ownership of health initiatives and cultural needs are recognized), these programs will not be as effectual.

Aboriginal people teach through the art of story telling, and an example of this is "Grandmother Law." The grandmothers teach the young girls about antenatal care through story telling whilst walking through bushland. I feel that the image and story on the cover of *Preventing Chronic Disease* (Volume 2: No. 4, Oct 2005) is a good reminder to health care providers to work side by side with the indigenous communities and respect each other's knowledge and stories. I have stuck it up on my office wall as a reminder to myself as well. I gave a copy to my Aboriginal colleagues, and they loved the cover's description because story telling is an important part of Aboriginal culture. I have enjoyed the journal and shared many of the articles with colleagues.

